# Advanced Heart Block and Asystole After Regadenoson Infusion: When Cautionary Tales Become Reality

**DOI:** 10.7759/cureus.50787

**Published:** 2023-12-19

**Authors:** Christopher S Park, Amin Nadeem

**Affiliations:** 1 Department of Internal Medicine, Chicago Medical School at Rosalind Franklin University of Medicine and Science, North Chicago, USA; 2 Section of Critical Care, Captain James Lovell FHCC (Federal Health Care Center) and Rosalind University of Medicine and Science, North Chicago, USA

**Keywords:** coronary artery disease, nuclear stress myocardial perfusion imaging, asystole, second-degree heart block, drug-related side effects and adverse reactions, regadenoson

## Abstract

Regadenoson (brand name Lexiscan) is a specific adenosine receptor agonist commonly used in pharmacologic stress testing due to its coronary vasodilatory effects. Despite it being generally well-tolerated, the American Society of Nuclear Cardiology established absolute and relative contraindications to the use of regadenoson in patients with certain co-morbidities such as uncontrolled hyper/hypotension, sinus node disease, and second-degree heart blocks. While cases of advanced heart block after the administration of regadenoson have been reported, they remain incidental. We report the case of an 84-year-old male sustaining second-degree type II heart block, followed by pulseless electrical activity and asystole after the administration of regadenoson.

## Introduction

Coronary artery disease (CAD) remains a leading cause of death in the USA. Cardiac stress testing remains the cornerstone of non-invasive diagnosis and stratification of suspected or known CAD, respectively [1,2]. While standard exercise treadmill testing remains the most cost-effective diagnostic approach [3], it lacks sensitivity when exercise intensity is inadequate (i.e., when heart rate fails to reach 85% of the maximal predicted heart rate) or in the presence of certain EKG abnormalities at baseline [1,3].

Single-photon emission computed tomography myocardial perfusion imaging (SPECT-MPI), also known as nuclear stress test imaging, is used to screen for and risk stratify patients with CAD in patients who cannot exercise adequately or have contraindications to exercise [4,5].

Regadenoson is a selective adenosine A2A receptor agonist that avoids activation of the A1 receptors on the AV node, reducing, in theory, the likelihood of AV block and bradycardia associated with adenosine [6]. The ADVANCE MPI (adenosine vs regadenoson comparative evaluation for myocardial perfusion imaging) phase 3 trial demonstrated the non-inferiority of regadenoson to adenosine [7]. Building upon the main trial, the ADVANCE MPI 2 trial showed virtually identical scintigraphic results in size and severity of left ventricular perfusion defects after quantitative analysis [8].

Adverse effect incidence noted during the ADVANCE MPI phase 3 trial included 2.8% of ADVANCE-MPI patients developing a first-degree AV block, one patient with a Wenckebach second-degree AV block, and no instances of complete heart block or asystole. Vindicated by these trials, improved side effect profile, and convenience of administration, regadenoson has largely supplanted adenosine and dipyridamole for SPECT-MPI around the world [9].

Here, we report a case of high-degree AV block followed by asystole after the administration of regadenoson in a patient with multiple risk factors for CAD as well as preexisting first-degree AV block and asymptomatic sinus bradycardia.

## Case presentation

An 84-year-old male patient with a medical history pertinent for obesity (body mass index 35 kg/m^2^), hypertension, 20 pack-year smoking, hyperlipidemia, type 2 diabetes mellitus, asymptomatic bradycardia, and obstructive sleep apnea presented for preoperative cardiac risk stratification for elective total knee arthroplasty. He was compliant with his home medications: atorvastatin, melatonin, multivitamin, and trazodone; it is important to note, however, that the patient was enrolled in a randomized controlled trial involving atorvastatin versus placebo.

A recent transthoracic echocardiogram demonstrated normal left ventricular function without wall motion abnormalities, ejection fraction of 65%, and grade I diastolic dysfunction with mild bi-atrial enlargement without significant valvular abnormality. Given the patient’s history of diabetes mellitus and diminished exercise tolerance of less than four metabolic equivalents of tasks (METs), he underwent an electrocardiogram (ECG)-gated regadenoson SPECT-MPI to assess for significant coronary artery stenosis per ACC/AHA (American College of Cardiology/American Heart Association) preoperative risk assessment guidelines [2]. Pertinent labs and findings prior to regadenoson SPECT-MPI are reviewed in Table 1.

**Table 1:** Pertinent labs and findings prior to regadenoson SPECT-MPI Out-of-reference range findings are marked with an asterisk (*). SPECT-MPI: single-photon emission computed tomography myocardial perfusion imaging

Characteristic	Value	Reference range
COVID-19 antigen	Negative	Negative
Hemoglobin A1C	6.1*	< 5.6 (%)
White blood cell count	6.0	4 – 11 k/µL
Hemoglobin	14.2	13 – 17 g/dL
Mean corpuscular volume	87.4	82 -97 fL
Platelet count	161	130 – 400 K/µL
Fasting glucose	106	70 – 99 mg/dL
Urea nitrogen	19	7 – 21 mg/dL
Creatinine	1.13	0.67 – 1.17 mg/dL
Estimated glomerular filtration rate (eGFR)	64	≥ 60 mL/min/1.7
Sodium	136	136 – 145 mmol/L
Potassium	4.3	3.5 – 4.7 mmol/L
Chloride	103	98 – 109 mmol/L
Carbon dioxide	28	20 – 31 mmol/L
Calcium	9.4	8.7 – 10.4 mg/dL
Vitamin D, 25-OH	135.9*	30 – 100 ng/mL
Total cholesterol	189	0 -200 mg/dL
Triglycerides	130	0 – 150 mg/dL
High-density lipoprotein (HDL)	47	≥ 40 mg/dL
Low-density lipoprotein (LDL)	116*	0 – 100 mg/dL
B12 vitamin	630	193 - 986
Electrocardiogram parameters		
Ventricular rate	75	60 – 100 bpm
Rhythm	Normal sinus rhythm	
PR interval	202*	120 – 200 ms
QRS duration	92	80 – 100 ms
QT, corrected	424	350 – 450 ms

**Figure 1:**
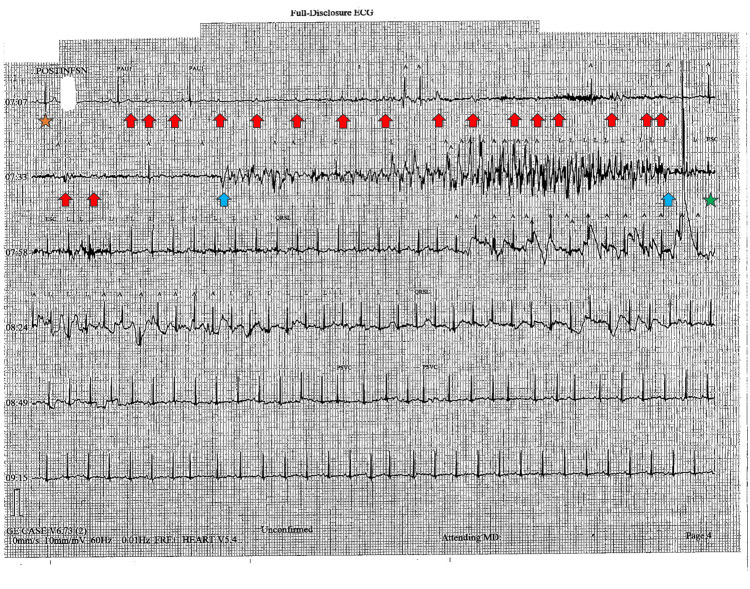
Single lead (II) ECG tracing during the transition from progressively worsening sinus bradycardia to third-degree AV block and asystole Orange star: Marks the transition from progressively worsening sinus bradycardia to 3rd degree heart block. This correlates to the second to last QRS complex in Figure 2, also marked with an orange star. Red arrows: Nonconducted p-waves, with no clear relationship between the number of p-waves and QRS complexes. Blue stars: Onset and cessation of compression artifacts marking CPR during activation of the Code Blue system. Green star: Return of spontaneous circulation. ECG: electrocardiogram; CPR: cardiopulmonary resuscitation

**Figure 2:**
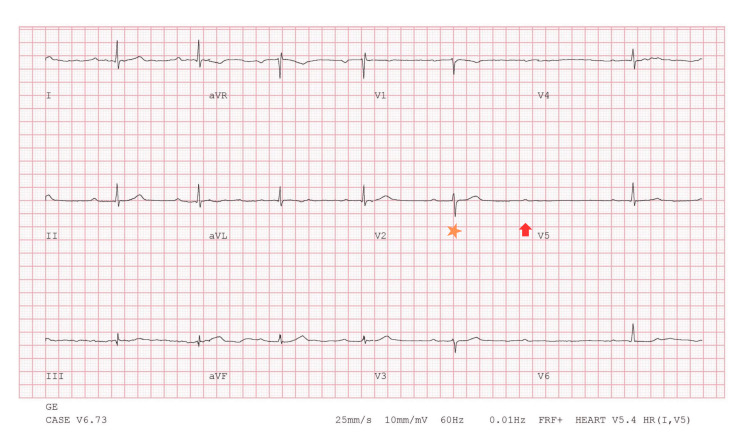
Twelve lead ECG seven minutes post-infusion demonstrating sinus bradycardia degrading to second-degree AV block immediately preceding the onset of third-degree AV block in Figure 1 Orange star: Transition from progressively worsening sinus bradycardia to second-degree type II AV block, corresponding to the orange star in Figure 1. Red arrow: Nonconducted P-wave. ECG: electrocardiogram

**Figure 3:**
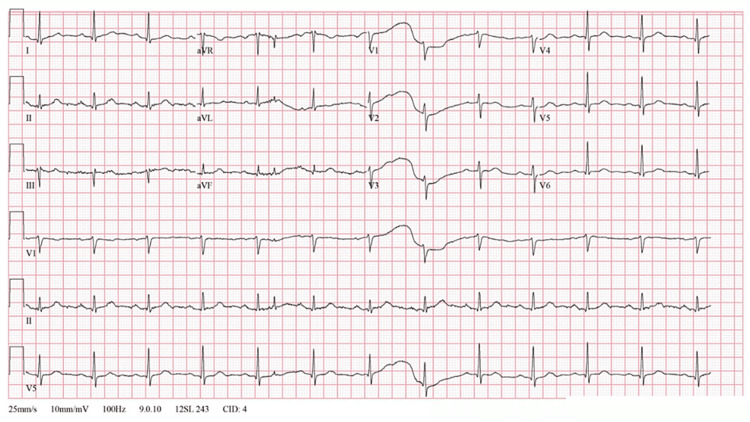
Baseline ECG one month prior to regadenoson SPECT-MPI showing first-degree AV block and sinus bradycardia ECG: electrocardiogram; SPECT-MPI: single-photon emission computed tomography myocardial perfusion imaging

**Figure 4:**
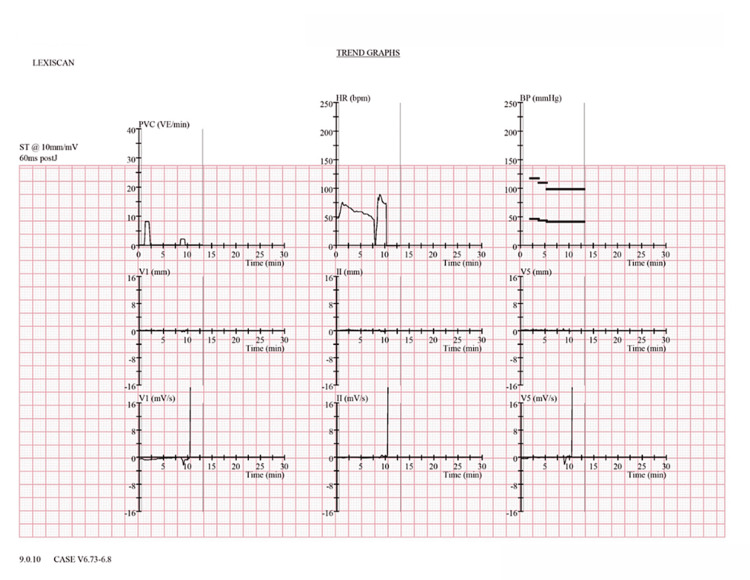
Concurrent ECG during regadenoson SPECT-MPI, with trend graphs showing the initial increase in heart and PVCs immediately post-infusion, with asystole near the seven-minute post-infusion mark ECG: electrocardiogram; SPECT-MPI: single-photon emission computed tomography myocardial perfusion imaging; PVC: premature ventricular contraction

**Figure 5:**
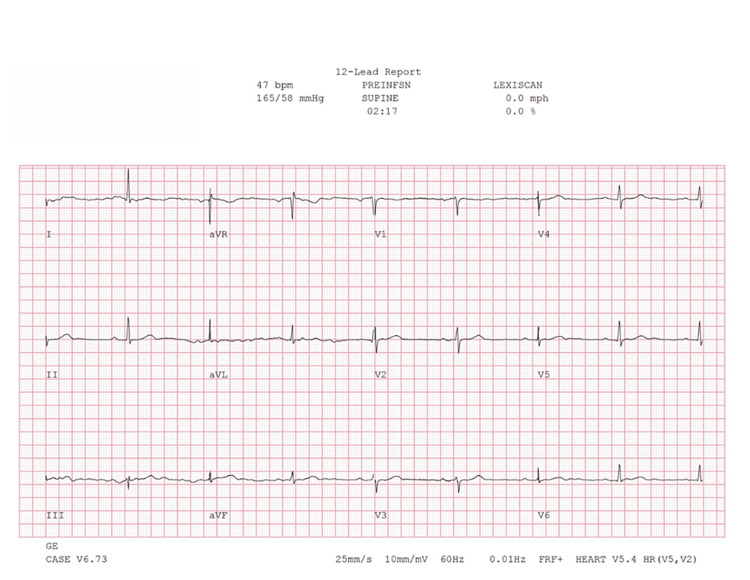
Concurrent ECG during regadenoson SPECT-MPI prior to regadenoson infusion demonstrating sinus bradycardia and first-degree AV block ECG: electrocardiogram; SPECT-MPI: single-photon emission computed tomography myocardial perfusion imaging

**Figure 6:**
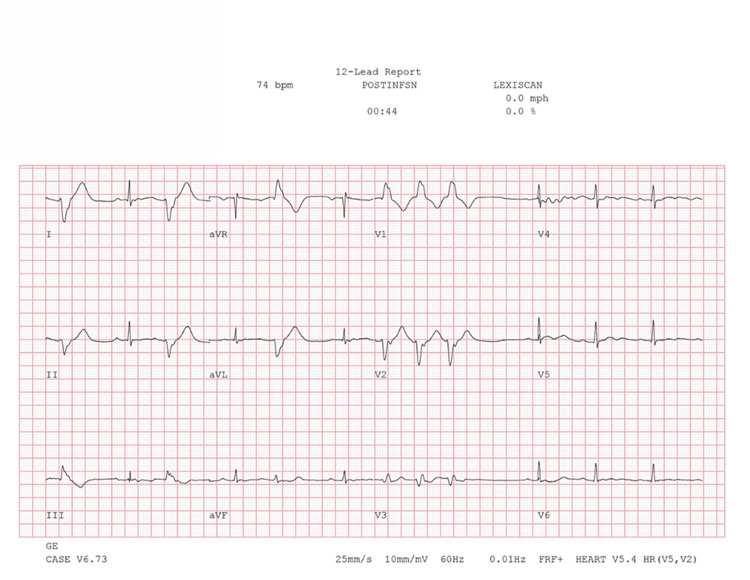
Concurrent ECG during regadenoson SPECT-MPI showing PVCs 45 seconds after infusion ECG: electrocardiogram; SPECT-MPI: single-photon emission computed tomography myocardial perfusion imaging; PVC: premature ventricular contraction

**Figure 7:**
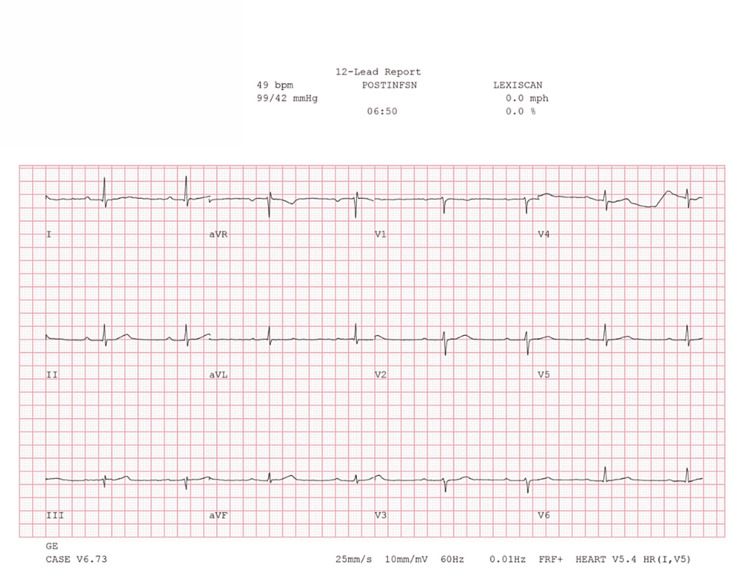
Concurrent ECG during regadenoson SPECT-MPI demonstrating return to baseline rhythm approximately 7 minutes post-infusion ECG: electrocardiogram; SPECT-MPI: single-photon emission computed tomography myocardial perfusion imaging

**Figure 8:**
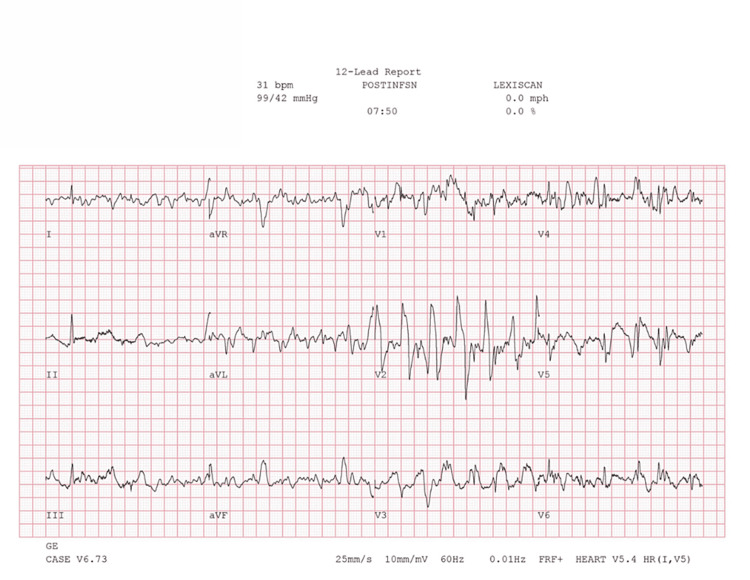
Concurrent ECG during regadenoson SPECT-MPI demonstrating compression artifacts during CPR ECG: electrocardiogram; SPECT-MPI: single-photon emission computed tomography myocardial perfusion imaging; PVC: premature ventricular contraction; CPR: cardiopulmonary resuscitation

Per protocol, 0.4 mg regadenoson was injected over 10-45 seconds, followed by technetium (99mTc) tetrofosmin radioisotope infusion. ECG initially showed sinus bradycardia without ST-segment abnormalities. No gastrointestinal or pre-syncopal symptoms were noted. The patient began to experience worsening baseline sinus bradycardia soon after regadenoson infusion, progressively worsening until the seven-minute post-infusion mark, whereupon the patient became unconscious and profoundly bradycardic. ECG revealed a new third-degree heart block that progressed to asystole within 15 seconds (Figure 1, Figure 2), triggering activation of code blue and cardiopulmonary resuscitation (CPR). Notably, this AV dissociation has intermittent narrow QRS complexes, suggesting a block at the AV node rather than at the Bundle of His or bundle-branch Purkinje system. Baseline ECG as well as concurrent ECG snapshots recorded during regadenoson SPECT-MPI are provided in Figures 3-8 for comparison.

Return of spontaneous circulation (ROSC) was achieved within 10 seconds of initiation of CPR, and the patient regained consciousness without disorientation or other signs of hypoxic brain injury. Atropine was not administered as the patient was hemodynamically stable and conscious with sinus tachycardia after ROSC. He was subsequently transferred to the intensive care unit (ICU) for observation and further evaluation.

In the ICU, high-sensitivity troponin was trended and found to be within normal range; repeat ECGs demonstrated normal sinus rhythm with pre-existing first-degree AV block, and continuous cardiac monitoring by telemetry was uneventful. The patient was scheduled for a dobutamine stress echocardiogram for preoperative cardiac risk stratification and discharged in good condition.

The patient later underwent a dobutamine stress echocardiogram, which showed no left ventricular dilation or inducible regional wall motion abnormalities and was otherwise negative for ischemia.

## Discussion

SPECT-MPI relies on the principle that when the myocardium is under stress, diseased tissue will receive less blood flow than normal heart muscle, with proportionately reduced uptake of gamma-radiation emitting isotopes, commonly thallium-201 or technetium-99m based [4]. Flow-limited stenosis in the coronary artery causes a relative reduction in blood flow during stress or vasodilation, with subsequently reduced radiotracer uptake and reflecting as a perfusing defect on SPECT-MPI. Vasodilators such as adenosine, dipyramidole, and regadenoson are used to induce coronary hyperemia and cardiac myocyte stress.

Adenosine is a key neuromodulator that affects cardiac function. It exerts its functions through G-protein-coupled adenosine receptors, ultimately modulating the activity of adenylyl cyclase and the pool of cytosolic cyclic adenosine monophosphate (cAMP). Adenosine receptors are divided into two main families, depending on the subtype of G-protein to which they are coupled. A1 receptors are coupled to the Gi protein and are thus inhibitory; they are expressed on cardiomyocytes as well as nodal cells in the heart. Stimulation of A1 receptors has a myocardial depressant effect through decreased cardiac automaticity, as well as delayed conduction of cardiac action potential. In contrast, A2 receptors are coupled to stimulatory Gs protein and are expressed on vascular endothelial and smooth muscle cells [10]. Stimulation of A2 receptors has a coronary vasodilatory effect, with minimal effect on cardiac automaticity or conduction of action potential.

Since its approval by the Food and Drug Administration (FDA) in 2008, regadenoson’s implied advantage has been its high specificity to A2A receptors, a subtype of A2 receptors while having negligible effects on A1 receptors (over 12 times more specific to A2A, compared to A1). This specificity would allow for maximal coronary dilation, without affecting cardiac rhythm or output. While generally better tolerated than its predecessors, regadenoson revealed a side-effect profile different from that of adenosine or dipyridamole.

One single-center, retrospective cohort study at a large community teaching hospital revealed that dipyridamole was associated with fewer adverse events than regadenoson in patients undergoing SPECT-MPI. This was primarily in regard to non-life-threatening complaints such as dyspnea, gastrointestinal upset, and chest pain. Regadenoson had more episodes of heart block at 3 to 1, but this finding was not statistically significant. In this case, these results prompted this institution to switch back to the much more cost-effective dipyridamole [6].

A comprehensive review of adverse effects by Andrikopoulou et al. [11] corroborates the reduced incidence of AV block of regadenoson compared to adenosine noted in the ADVANCED-MPI trial, with a total of 56 cases of third-degree heart block and 26 cases of sinus arrest reported via the FDA Adverse Event Reporting System (FAERS) as of June 2017, and noting the incidence of a high-degree AV block (second or third-degree AV block) related to regadenoson administration during SPECT-MPI being observed less frequently at 0.3% compared to 8.58% for adenosine [11].

Brinkert et al. reported on seven to eight patients who developed vasovagal responses preceded by nausea and retching, two episodes of asystole, and other frequent but mild symptoms, with dyspnea and chest pain being the most common. They also reported seven episodes of symptomatic hypotension with inappropriate bradycardia, which they assessed to be vasovagal in etiology, with two progressing to sinus arrest and asystole [9].

In a similar vein, a case series of three incidents of asystole and severe bradycardia proposed that sympathetic activation of the nucleus tractus solitarii and hypothalamus in the setting of significant gastrointestinal distress, particularly nausea, retching, and vomiting, could mediate via the A2A receptor agonism potential bradycardia by inducing intense vagal stimulation [12].

Still, the mechanism by which AV block occurs is not known though the aforementioned authors posited the hypothesis that they result from a combination of a prominent vasovagal reaction or regadenoson-induced release of endogenous adenosine. In the latter scenario, patients with severe CAD or extensive collateral circulation may experience coronary steal or a sudden drop in systemic blood pressure and subsequent ischemia, which may lead to the overproduction of endogenous adenosine and activation of A1 receptors in the SA and AV nodes.

Our patient experienced no gastrointestinal symptoms or any other complaints prior to the onset of bradycardia, unlike the cases reported in Brinkert et al. [9], and likely did not induce bradycardia via intense vagal stimulation. In addition, while they did have several risk factors for coronary artery disease, he later underwent a dobutamine stress echocardiogram that showed no evidence of symptomatic coronary artery disease, making the endogenous release of adenosine in the setting of reversible ischemia unlikely. We speculate that the patient may have expressed a variant of the adenosine A1 receptor that could bind regadenoson with greater affinity and induce bradycardia and AV block. Given that screening for such polymorphisms in phenotypical expression prior to SPECT-MPI may not be cost-effective, it may be more prudent to consider clinical contraindications prior to regadenoson infusion. In this case, the patient had preexisting first-degree AV block and sinus bradycardia, which may potentially be associated with these polymorphisms.

Current guidelines list second or third-degree AV block, as well as sinus node disease, as absolute contraindications to the usage of regadenoson. Our patient had a borderline first-degree AV block and did not meet any of the absolute or relative contraindications for the use of regadenoson as noted by Henzlova et al. [13]. While high-grade heart block and/or asystole have previously been reported with regadenoson [14-16], our work serves as a reminder that this remains a rare but possible complication (≈ 0.05% occurrence rate) [12] requiring close surveillance during the procedure and preparedness to intervene by Advanced Cardiovascular Life Support (ACLS) trained staff, especially if a history of first-degree AV block or sinus bradycardia is present.

## Conclusions

Regadenoson is an adenosine receptor agonist commonly used in pharmacologic stress testing. Its improved specificity compared to its predecessors adenosine and dipyridamole gives it a much better tolerated side effect profile. Guidelines caution against its use in patients with a preexisting high-degree AV block or sinus node disease, though as our case report demonstrates, this rare complication can occur in patients without these contraindications. AV block and asystole are noted to be primarily in patients with gastrointestinal distress. Therefore, a high index of suspicion for this rare complication and a readiness to intervene by ACLS-trained staff, especially in those with gastrointestinal distress, first-degree AV block, or sinus bradycardia, are warranted for all patients undergoing SPECT-MPI with regadenoson-based pharmacologic stress testing.
